# Reduced DNA methylation at the *PEG3 DMR* and *KvDMR1* loci in children exposed to alcohol *in utero*: a South African Fetal Alcohol Syndrome cohort study

**DOI:** 10.3389/fgene.2015.00085

**Published:** 2015-03-10

**Authors:** Matshane L. Masemola, Lize van der Merwe, Zané Lombard, Denis Viljoen, Michèle Ramsay

**Affiliations:** ^1^Division of Human Genetics, National Health Laboratory Service, School of Pathology, Faculty of Health Sciences, University of the Witwatersrand, Johannesburg, South Africa; ^2^Department of Statistics, Faculty of Natural Sciences, University of the Western Cape, Cape Town, South Africa; ^3^Molecular Biology and Human Genetics, Faculty of Medicine and Health Sciences, Stellenbosch University, Cape Town, South Africa; ^4^Sydney Brenner Institute for Molecular Bioscience, University of the Witwatersrand, Johannesburg, South Africa; ^5^School of Molecular and Cell Biology, Faculty of Science, University of the Witwatersrand, Johannesburg, South Africa; ^6^Foundation for Alcohol Related Research, Cape Town, South Africa

**Keywords:** fetal alcohol syndrome, imprinted genes, epigenetics, *PEG3*, *KvDMR1*, *H19 ICR*, *IG-DMR*

## Abstract

Fetal alcohol syndrome (FAS) is a devastating developmental disorder resulting from alcohol exposure during fetal development. It is a considerable public health problem worldwide and is characterized by central nervous system abnormalities, dysmorphic facial features, and growth retardation. Imprinted genes are known to play an important role in growth and development and therefore four imprinting control regions (ICRs), *H19 ICR*, *IG-DMR*, *KvDMR1* and *PEG3 DMR* were examined. It is proposed that DNA methylation changes may contribute to developmental abnormalities seen in FAS and which persist into adulthood. The participants included FAS children and controls from the Western and Northern Cape Provinces. DNA samples extracted from blood and buccal cells were bisulfite modified, the ICRs were amplified by PCR and pyrosequencing was used to derive a quantitative estimate of methylation at selected CpG dinucleotides: *H19 ICR* (six CpG sites; 50 controls and 73 cases); *KvDMR1* (7, 55, and 86); *IG-DMR* (10, 56, and 84); and *PEG3 DMR* (7, 50, and 79). The most profound effects of alcohol exposure are on neuronal development. In this study we report on epigenetic effects observed in blood which may not directly reflect tissue-specific alterations in the developing brain. After adjusting for age and sex (known confounders for DNA methylation), there was a significant difference at *KvDMR1* and *PEG3 DMR*, but not the *H19 ICR*, with only a small effect (0.84% lower in cases; *p* = 0.035) at *IG-DMR*. The two maternally imprinted loci, *KvDMR1* and *PEG3 DMR*, showed lower average locus-wide methylation in the FAS cases (1.49%; *p* < 0.001 and 7.09%; *p* < 0.001, respectively). The largest effect was at the *PEG3 DMR* though the functional impact is uncertain. This study supports the role of epigenetic modulation as a mechanism for the teratogenic effects of alcohol by altering the methylation profiles of imprinted loci in a locus-specific manner.

## INTRODUCTION

Alcohol is a potent teratogen with devastating effects on the developing fetus. The most profound effects of prenatal alcohol exposure are on neuronal development, resulting in adverse cognitive and behavioral outcomes with lifelong implications, distinct dysmorphic features (shortened palpebral fissures, smooth philtrum, and thin vermilion border to the upper lip), and pre- and postnatal growth retardation (Stratton et al., 1996; Riley and McGee, 2005; Floyd et al., 2006). The outcomes are collectively referred to as fetal alcohol spectrum disorders (FASD) and range in severity with fetal alcohol syndrome (FAS) at the most severe end of the spectrum (Sokol et al., 2003). FAS is the leading cause of preventable mental retardation and developmental disability in the world. It is an international problem that shows no racial boundaries (Clarren and Smith, 1978; Masotti et al., 2006) and the consequences of prenatal alcohol exposure represent a major public health problem worldwide (May and Gossage, 2001; Sokol et al., 2003; Riley et al., 2011).

The worldwide average prevalence of FAS is estimated at 0.97 per 1000 live births, yet in some communities it is much higher (Abel and Hannigan, 1995; May and Gossage, 2001; McKinstry, 2005). Notably, the prevalence of FAS in South Africa is one of the highest reported in the world, at 40.5–46.4 per 1000 children of school going age in the Western Cape Province (May et al., 2000), confirmed in two follow up studies from the same area reporting an increasing prevalence of 65.2–74.2 (Viljoen et al., 2005) and 68–89.2 per 1000 (May et al., 2007). In addition a study in the Northern Cape Province reported a similar prevalence of 67.2 per 1000 (Urban et al., 2008).

Fetal alcohol syndrome is a complex multifactorial condition and although prenatal alcohol exposure is the primary trigger, twin concordance studies and animal models suggest a significant genetic susceptibility for the development of FAS (Streissguth and Dehaene, 1993; Becker et al., 1996). Recent studies have proposed an epigenetic etiology and supporting evidence for such a mechanism is accumulating (Garro et al., 1991; Haycock, 2009; Ungerer et al., 2013). Gene expression disturbances can be caused by changes in DNA methylation, molecular modification of histones and through RNA interference. These mechanisms work together to produce a unique, and reversible epigenetic signature that regulates gene expression through chromatin remodeling. DNA methylation has been investigated extensively as a mechanism of alcohol teratogenesis.

Genomic imprinting is an epigenetic phenomenon resulting in mono-allelic gene expression according to the parent of origin in a locus-specific manner. It is mediated by differential DNA methylation and imprinted loci play an important role during normal development (Jirtle et al., 2000; Rodenhiser and Mann, 2006). The DNA methylation status can be influenced by the environment leading to a functional impact mediated by changes in the epigenome (Jirtle and Skinner, 2007). Imprinted genes are therefore suitable candidates for investigating the effects of teratogens on disease etiology. Almost all imprinted genes contain differentially methylated regions (DMRs) which serve as a mark that differentiates the paternal allele from the maternal allele. Some DMRs which regulate the methylation patterns of a cluster of imprinted genes are referred to as primary DMRs or imprinting control regions (ICRs). The CpG methylation at ICRs is established in the gametes and maintained in somatic tissues of offspring throughout development (Smallwood and Kelsey, 2012). Despite this trend, they may still be subject to tissue-specific effects and extrapolation from the tissue under investigation should be done with care. On the other hand, the imprinting of secondary DMRs is established after fertilization (Geuns et al., 2007). Individual loci may be hyper- or hypomethylated following alcohol exposure. A study by Kaminen-Ahola et al. (2010) reported that maternal alcohol exposure tended to induce hypermethylation at the A^vy^ locus, while Haycock and Ramsay (2009) reported hypomethylation at the *H19 ICR* in mouse placenta following *in utero* alcohol exposure and Stouder et al. (2011) also showed hypomethylation at the *H19 ICR* in the brain and sperm of *in utero* exposed offspring (Stouder et al., 2011). A study by Liu et al. (2009) has demonstrated that alcohol exposure during early neurulation can induce aberrant changes in DNA methylation with associated changes in gene expression in mice. These studies support an epigenetic mechanism as a contributing factor for the development of features observed in FASD. It is widely suggested that the effect is mediated through the interruption of the one carbon pathway that is critical in production of the methyl groups in the maintenance of DNA methylation (Halsted et al., 2002; Liu et al., 2009). Alcohol exposure is correlated with reduced DNA methylation through several plausible mechanisms. Firstly acetaldehyde, a metabolite of alcohol metabolism, inhibits methyltransferase activity, and secondly, folate deficiency as a result of alcohol consumption and poor nutrition, reduces the pool of methyl donors.

In this study we examined quantitative changes in DNA methylation in blood and buccal cells from individuals with FAS, compared to unaffected controls, at four ICRs that regulate gene expression at loci that are important during fetal growth and development: *H19 ICR*, *KvDMR1, IG-DMR,* and *PEG3 DMR*.

## MATERIALS AND METHODS

### STUDY PARTICIPANTS AND SAMPLE COLLECTION

The study participants included 87 individuals with a diagnosis of FAS and 58 controls. All participants were of mixed ancestry, referred to as “Coloreds” in the South African context, and were resident in the Western Cape and Northern Cape provinces of South Africa. The FAS cases were recruited from Wellington in the Western Cape and De Aar and Upington in the Northern Cape. They were diagnosed by a team of trained clinicians from the Division of Human Genetics, NHLS, Braamfontein, Johannesburg, and also the Foundation for Alcohol Related Research (FARR; http://www.farr-sa.co.za), led by Denis Viljoen. The control participants were recruited from the Northern Cape and no phenotype data were collected. The cases and controls were not age matched. The FAS cases has a median age of 9 years (range 1–16 years) and the control participants were 17–26 years of age (median age 20 years). Adult participants provided informed consent and the parents or guardians of minor participants provided informed consent on their behalf. Ethics approval for the study was obtained from the University of the Witwatersrand Committee for Research on Human Subjects (Medical) (Protocol numbers M02/10/41, M03/10/20 and M080548). Venous blood samples were collected into EDTA by qualified phlebotomists and buccal swabs were collected by the research staff.

### DNA EXTRACTION FROM BLOOD AND BUCCAL TISSUES

DNA was extracted from whole blood using a manual salting out method according to a modified protocol from Miller et al. (1988). The buccal tissue DNA was extracted using the Gentra Puregene buccal cell kit (Qiagen, Valencia, CA, USA).

### DNA BISULFITE MODIFICATION AND PCR AMPLIFICATION

Genomic DNA was bisulfite modified using the EZ-DNA Methylation Gold Kit^™^ (Zymo Research, Orange, CA, USA). Published primer sets and custom designed primer sets were used to amplify specific regions within the ICRs of four imprinted loci: *H19 ICR; IG-DMR*; *KvDMR1*; and *PEG3 DMR*. Each locus is described briefly and the details of the PCR and sequencing primers are shown in Table 1.

**TABLE 1:** **Locus specific information for PCR amplification and pyrosequencing**.

**Locus (contig)**	**No. CpG sites**	**PCR primers (5′-3′)**	**Amplicon length (bp)**	**Annealing Temp (°C)**	**Sequencing primer (5′-3′)**	**Reference**
*H19 ICR* (AF087017)	6	Outer reverse CTTAAATCCCAAACCATAACACTA	217	61.5	TGGTTGTAGTTGTGGAAT	Present study
		Outer forward GTATATGGGTATTTTTTGGAGGT				
		Inner forward GTATATGGGTATTTTTTGGAGGT				
		Inner reverse Tag-ATATCCTATTCCCAAATAA		53		
*IG-DMR* (A117190)	7	Forward Tag-TTTATTGGGTTGGGTTTTGTTAG	267	58	Primer 1 CAATTACAATACCACAAAAT	Present study
	3	Reverse AACCAATTACAATACCACAAAATT			Primer 2 CCATAAACAACTATAAACCT	Present study
*KvDMR1* (U90095)	7	Forward TTAGTTTTTTGYGTGATGTGTTTATTA	101	55	TTGYGTGATGTGTTTATTA	Bourque et al. (2010)
		Reverse Tag-CCCACAAACCTCCACACC				
*PEG3 DMR* (AC006115)	7	Reverse Tag-CCTATAAACAACCCCACACCTATAC	272	62	GGGGGTAGTTGAGGTT	Boissonnas et al. (2010)
		Forward TAATGAAAGTGTTTGAGATTTGTTG				

Tag-5′-biotin-GACGGGACACCGCTGATCGTTTA-3′—universal biotin labeled tag.

The pre-pyrosequencing PCR step requires that one of the primers is 5′ biotin labeled. In this study we used a universal biotin labeled primer (5′-biotin-GACGGGACACCGCTGATCGTTTA-3′) which was included in the PCR cocktail to generate labeled DNA fragments (Colella et al., 2003). The sequences of primers that were designed to be biotin labeled therefore had a 23 bp complementary tag sequence added to their 5′ ends for the priming of the universal biotin labeled primer. These primers are shown in Table 1 as “tag” primers. Unless specified to the contrary, primer sets were designed using the PSQ assay design software (Biotage, Uppsala country, Sweden).

The *H19 ICR* contains seven CTCF binding sites, of which the sixth is differentially methylated. The sixth CTCF binding site was the target region in this study and contains five CpGs, but the amplified region included one extra CpG which was also included in the analysis. For the *H19 ICR* amplification, nested PCR was used with an outer and an inner PCR primer sets. The PCR reactions for this region were performed in triplicate.

The amplified *IG-DMR* region contains 15 CpGs, but only 10 CpGs were analyzed using two different sequencing primers (1 and 2), where one analyzed three and the other analyzed seven CpG sites. PCR primers used for amplification of the *KvDMR1* are published primers and the amplicon contains seven CpGs, including a differentially methylated *NotI* site (Bourque et al., 2010). The PCR forward primer and pyrosequencing sequencing primer had a wobble introduced to accommodate an unavoidable CpG site in the sequence template that could either be methylated or unmethylated. The *PEG3 DMR* amplified region contains 14 CpGs but only seven CpGs were analyzed. The PCR assays for *IG-DMR*, *KvDMR1*, and *PEG3 DMR* were run in duplicate.

### PYROSEQUENCING FOR QUANTIFICATION OF DNA METHYLATION ANALYSIS

DNA methylation of the different amplified ICRs was quantified by pyrosequencing using the PSQ 96MA system with the Pyro- Gold SQA reagent kit (Biotage, Uppsala, Uppsala country, Sweden). Pyrosequencing assays and sequencing primers (Table 1) were designed using PSQ Assay Design Software and the sequencing was done in triplicate (*H19 ICR*) or duplicate (*IG-DMR*, *KvDMR1*, and *PEG3 DMR*). The percentage methylation for each of the CpG sites within the target region was calculated using Pyro Q-CpG software (Biotage, Uppsala, Uppsala country, Sweden). Two non-CpG cytosine bases were included in all the pyrosequencing assays as internal controls to assess successful bisulfite conversion. Samples containing >5% unsuccessfully converted non-CpG cytosines were discarded.

### STATISTICAL ANALYSIS

We analyzed methylation data for 145 individuals, 87 FAS cases, 58 controls. Not every individual provided complete data. There was no age overlap due to the cases being of primary school age (younger than 17 years old, mean age 9 years) and the controls being 17 years or older. This means that the age effect (difference between young and older) cannot be distinguished from the fetal alcohol (case-control) effect in this study. However, the effect per additional year of age could be estimated within each group. Both groups had similar gender distributions, as summarized in Table 2.

**TABLE 2:** **Summary table for number of samples, sex and age distribution for the different loci tested in the control and case groups**.

	**Controls**				**Cases**			
		**Age**	**Sex**			**Age**	**Sex**	
**Locus**	***N***	**Mean (years) (min:max)**	**Male**	**Female**	***N***	**Mean (years) (min:max)**	**Male**	**Female**
*H19 ICR*	50	21 (17:26)	27	23	73	8.7 (1:16)	41	32
*KvDMR1*	55	21 (17:26)	27	28	86	8.4 (1:16)	46	40
*IG-DMR*	56	21 (17:26)	29	27	84	8.5 (1:16)	45	39
*PEG3 DMR*	50	21 (17:26)	25	25	79	8.7 (1:16)	46	33

Linear mixed-effects models were used to generate all the results reported here. These analyses are based on joint models, where all the original methylation observations (individual replicates) are put into a single model to simultaneously do the tests. One advantage is that it avoids some false positive results, because all the results are adjusted for each other. These models also enabled us to adjust for different kinds of random variation as random effects: that between sites, and that between individuals, and that within individuals (between replicates). Adjusting for the variation between individuals is a different way of saying we adjusted for the correlation between replicates on the same individual. After confirming, using linear mixed-effects models, that age and sex were confounders, all further models were adjusted for them, as fixed effects. All *p*-values, effects sizes and standard errors (SE) come from interaction terms in the models. All results corresponding to *p*-values below 0.05 are described as significant, below 0.01 as highly significant and below 0.001 as very highly significant.

The observed methylation data are summarized with box plots. Each box extends from the first quartile to the third quartile (interquartile range), the line inside the box is at the median, and the whiskers extend to the non-outlying minimum and maximum, respectively. Outliers are shown as open circles. The freely available environment for statistical computing and graphics, R (R Core Team, 2014), and R package (Pinheiro et al., 2015), were used for these analyses.

## RESULTS

The 87 FAS cases were recruited from several areas of the Western Cape and the Northern Cape, whereas the 58 control participants were mainly recruited from the Northern Cape. There are differences in the numbers of individuals tested per locus, due to failure to amplify in specific samples for specific loci. Similar percentages per sex were tested, 30 (52%) males and 28 (48%) females in the controls and 47 (54%) males and 40 (46%) females in the cases. The control participants (*N* = 58) all donated blood samples and of the 87 FAS cases, eight donated buccal samples and the remainder donated blood. A summary for the number of samples, sex and age distribution at the different loci in the case and control groups is shown in Table 2.

To address tissue specificity of DNA methylation at an imprinted locus, we showed that there was no significant difference in percentage methylation at the *H19 ICR* locus CpG sites between buccal and blood samples from 50 random participants from another study (data not shown). Methylation status between the two tissues was not assessed at *KvDMR1*, *IG-DMR*, and *PEG3 DMR*. Based on two previous studies, we concluded that methylation profiles at these ICRs are unlikely to differ between the two tissues. Bourque et al. (2010) compared average methylation profiles at *KvDMR1* between blood and saliva tissues in healthy adults and reported that their methylation patterns were similar. In addition Woodfine et al. (2011) examined the methylation patterns of 17 germline DMRs (including *H19 ICR*, *KvDMR1*, *IG-DMR*, and *PEG3 DMR*) amongst several somatic tissues (including brain, breast, colon, heart, kidney, and liver) and reported that the average methylation did not vary amongst the tissues. It is therefore unlikely that the origin of the tissue for the DNA methylation studies is a significant confounder in this study.

**FIGURE 1:**
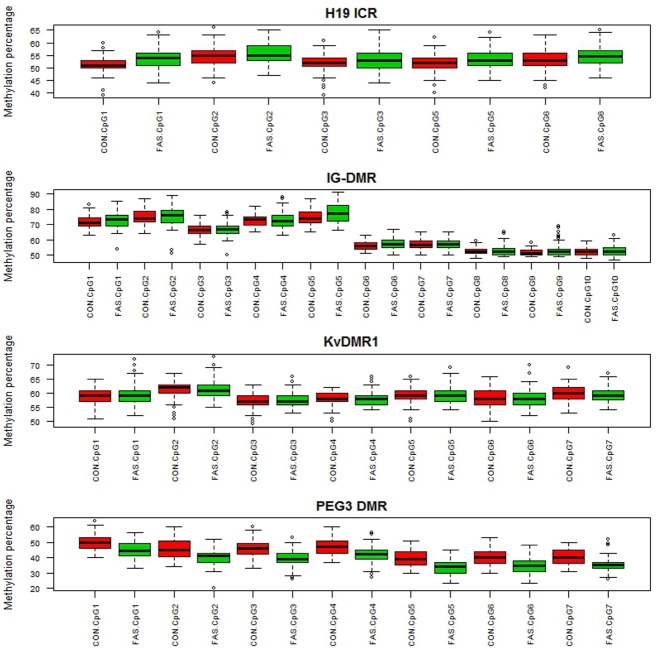
**Boxplots summarizing the observed percentage methylation at the CpG sites in *H19 ICR*, *IG-DMR*, *KvDMR1*, and *PEG3 DMR* in controls (CON) and cases (FAS)**.

**FIGURE 2:**
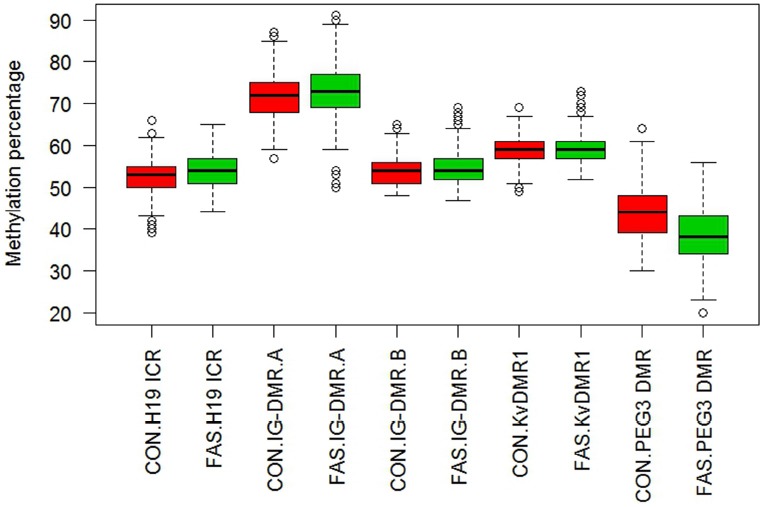
**Boxplot of observed percentage methylation per locus, *H19 ICR*, *IG-DMR*, *KvDMR1*, and *PEG3 DMR*, in controls (CON) and cases (FAS).** A significant difference was detected for *H19 ICR* between cases and controls (*p* = 0.024), but after adjustment for sex and age this was no longer significant. The estimated methylation percentage difference between cases and controls at *PEG3 DMR* was highly significant (*p* < 0.001) and remained so after adjustment for age and sex.

Figure 1 contains box plots summarizing the observed percentage methylation at individual CpG sites at all loci (*H19 ICR*, *KvDMR1*, *IG-DMR*, and *PEG3 DMR*), in controls (CON) and cases (FAS). Figure 2 contains box plots summarizing the observed percentage average methylation at each locus: *H19 ICR*, *KvDMR1*, *IG DMR*, and *PEG3 DMR*, in controls (CON) and cases (FAS). It is not possible to visualize the data after correction for age and sex.

### AGE AND SEX AS POTENTIAL CONFOUNDERS IN DNA METHYLATION STUDIES ON IMPRINTED LOCI

Age and sex are reported confounders in DNA methylation studies and their effects were investigated in the present study. The results for sex are summarized in Table 3 and for age in Table 4. The sex effect was highly significant at *PEG3 DMR* in FAS cases, where males had an estimated 1.11% more methylation than females. In contrast, in controls at *PEG3 DMR*, males had a significant estimated 0.84% lower methylation compared to that in females. However estimated methylation did not differ by sex in control nor in FAS cases at any of *H19 ICR*, *IG-DMR.A*, *IG-DMR.B*, and *KvDMR1*. Since there was a significant difference at one locus, sex was adjusted for in downstream analyses. It was observed that *IG-DMR* has a wide variability in methylation at the different CpG sites analyzed. Most of the individuals had methylation of above 70% at CpG 1–5 while CpG site 6–10 have methylation of about 50%. Therefore *IG-DMR* was split into two regions for this analysis: sites 1–5 called *IG-DMR.A* and sites 6–10 called *IG-DMR.B.*

**TABLE 3:** **Comparison of methylation within a locus between sexes, separately in FAS and controls**.

**Locus**	**Group**	**Effect**	**SE**	***P*-value**
*H19 ICR*	CON	0.33	0.48	0.495
*H19 ICR*	FAS	–0.16	0.40	0.687
*IG-DMR1.A*	CON	0.37	0.46	0.428
*IG-DMR1.A*	FAS	0.18	0.38	0.626
*IG-DMR1.B*	CON	–0.45	0.46	0.329
*IG-DMR1.B*	FAS	–0.60	0.38	0.112
*KvDMR1*	CON	0.10	0.39	0.795
*KvDMR1*	FAS	–0.22	0.32	0.490
*PEG3 DMR*	CON	–0.84	0.41	0.042
*PEG3 DMR*	FAS	1.11	0.33	0.001

CON, controls; FAS, FAS case; Effect, the estimated percentage difference in methylation between males and females in the specific group at the specific locus, using linear mixed-effects models, as described in methods section; SE, standard error of the effect estimate. Significant: p < 0.05. Analyses are adjusted for variation between sites and also for variation between individuals and within individuals as random effects.

**TABLE 4:** **The estimated effect of 1 year of age on % methylation per locus per group**.

**Locus**	**Group**	**Effect**	**SE**	***P*-value**
*H19 ICR*	CON	–0.05	0.10	0.634
*H19 ICR*	FAS	0.02	0.06	0.749
*IG-DMR.A*	CON	0.02	0.10	0.861
*IG-DMR.A*	FAS	–0.43	0.06	<0.001
*IG-DMR.B*	CON	0.01	0.10	0.920
*IG-DMR.B*	FAS	–0.38	0.06	<0.001
*KvDMR1*	CON	0.19	0.08	0.016
*KvDMR1*	FAS	–0.11	0.05	0.021
*PEG3 DMR*	CON	–0.22	0.08	0.008
*PEG3 DMR*	FAS	0.00	0.05	0.948

CON, controls; FAS, FAS case; Effect, estimated percentage difference in methylation between patients of a specific age and those 1 year younger, in the specific group at the specific locus, using linear mixed-effects models, as described in methods section; SE, standard error of the effect. Significant: p < 0.05. Analysis is adjusted for sex (fixed), CpG sites, individuals and replicates (random effects).

Due to ethical considerations in the selection of control participants, the study design was sub-optimal in terms of age. All cases were below 17 years of age and all controls were 17 years and above, where the latter were able to give individual informed consent, but the parents or guardians consented to the participation of the cases. This means that age is strongly confounded and that it is not possible to tell whether any differences between cases and controls are caused by the age difference or not. However, the effect of age inside each of the groups could and was investigated.

Table 4 shows estimates of the difference in methylation percentage over 1 year of age, together with its SE and *p*-values in cases and controls.

The largest effects are seen at *IG-DMR.A* and *IG-DMR.B* in FAS cases, where the estimated methylation percentage decreased by 0.43 and 0.38% respectively, for a 1 year increase in age. At *KvDMR1*, for every year increase in age, there is a significant estimated methylation increase of 0.19% in controls but in FAS cases there is a significant decrease by 0.11%. Again the highly significant effect is seen at *PEG3 DMR* in the control group, where estimated methylation percentage decreases by 0.22% for every year increase in age. No age effect was observed at *H19 ICR* (either in cases or controls), nor at *IG-DMR.A* and *IG-DMR.B* (in controls) nor at *PEG3 DMR* (in cases). Table 5 summarizes, for each CpG site, the effect of 1 year of age on methylation, separately for controls and FAS cases, as well as the estimated difference between cases and controls in that effect. There are five CpG sites in *IG-DMR*, one in *KvDMR1*, where the effect of age on methylation is significantly lower in FAS cases and controls. At *IG-DMR* sites 2, 5, 6, 8, and 9, as well as at *KvDMR1* site 6, methylation decreased highly significantly with age in FAS cases but no significant effect was detected in controls. In *PEG3 DMR* site 2, the effect was significantly higher in FAS cases than controls.

**TABLE 5:** **The estimated effect of 1 year of age on % methylation per CpG site per locus**.

		**Control group**			**FAS cases**			**Estimated difference in age effect on methylation between FAS and CON**		
**Locus**	**Site**	**Age effect**	**SE**	***P*-value**	**Age effect**	**SE**	***P*-value**	**FAS-CON**	**SE**	***P*-value**
*H19 ICR*	CpG1	0.02	0.16	0.890	0.03	0.10	0.758	0.01	0.19	0.962
*H19 ICR*	CpG2	0.04	0.16	0.811	0.06	0.10	0.577	0.02	0.19	0.924
*H19 ICR*	CpG3	–0.16	0.16	0.298	0.02	0.10	0.851	0.18	0.19	0.326
*H19 ICR*	CpG5	0.10	0.16	0.522	–0.01	0.10	0.896	–0.11	0.19	0.541
*H19 ICR*	CpG6	–0.09	0.16	0.546	0.08	0.10	0.448	0.17	0.19	0.360
*IG-DMR*	CpG1	–0.05	0.17	0.789	–0.43	0.10	<0.001	–0.38	0.20	0.052
*IG-DMR*	CpG2	–0.19	0.17	0.273	–0.72	0.10	<0.001	–0.53	0.20	0.007
*IG-DMR*	CpG3	0.19	0.17	0.256	–0.02	0.10	0.811	–0.22	0.20	0.273
*IG-DMR*	CpG4	0.17	0.17	0.311	–0.17	0.10	0.089	–0.35	0.20	0.081
*IG-DMR*	CpG5	–0.11	0.17	0.520	–0.95	0.10	<0.001	–0.84	0.20	<0.001
*IG-DMR*	CpG6	–0.02	0.17	0.922	–0.45	0.10	<0.001	–0.43	0.20	0.030
*IG-DMR*	CpG7	0.05	0.17	0.774	–0.25	0.10	0.016	–0.30	0.20	0.134
*IG-DMR*	CpG8	0.03	0.17	0.878	–0.44	0.10	<0.001	–0.47	0.20	0.019
*IG-DMR*	CpG9	–0.04	0.17	0.812	–0.52	0.10	<0.001	–0.48	0.20	0.016
*IG-DMR*	CpG10	–0.03	0.17	0.863	–0.40	0.10	<0.001	–0.37	0.20	0.059
*KvdMR*	CpG1	0.26	0.17	0.122	–0.11	0.10	0.255	–0.37	0.20	0.056
*KvdMR*	CpG2	0.11	0.17	0.513	–0.04	0.10	0.687	–0.15	0.20	0.443
*KvdMR*	CpG3	0.10	0.17	0.562	–0.14	0.10	0.148	–0.24	0.20	0.218
*KvdMR*	CpG4	0.14	0.17	0.412	–0.09	0.10	0.363	–0.23	0.20	0.243
*KvdMR*	CpG5	0.26	0.17	0.126	–0.03	0.10	0.769	–0.29	0.20	0.142
*KvdMR*	CpG6	0.18	0.17	0.284	–0.22	0.10	0.023	–0.40	0.20	0.038
*KvdMR*	CpG7	0.15	0.17	0.380	–0.12	0.10	0.225	–0.27	0.20	0.170
*PEG3 DMR*	CpG1	–0.19	0.17	0.272	0.02	0.10	0.818	0.22	0.20	0.289
*PEG3 DMR*	CpG2	–0.28	0.17	0.115	0.25	0.10	0.017	0.53	0.20	0.010
*PEG3 DMR*	CpG3	–0.03	0.17	0.858	0.09	0.10	0.376	0.12	0.20	0.543
*PEG3 DMR*	CpG4	–0.11	0.17	0.523	0.03	0.10	0.793	0.14	0.20	0.495
*PEG3 DMR*	CpG5	–0.22	0.17	0.201	–0.14	0.10	0.194	0.09	0.20	0.665
*PEG3 DMR*	CpG6	–0.19	0.17	0.284	–0.16	0.10	0.125	0.03	0.20	0.895
*PEG3 DMR*	CpG7	–0.37	0.17	0.033	0.03	0.10	0.799	0.40	0.20	0.050

Effect is the estimated percentage difference in methylation between individuals of a specific age and those 1 year younger, in the specific group at the specific locus, using linear mixed-effects models, as described in methods section.

In light of these differences, sex and age were adjusted for in the subsequent analyses to assess differences between FAS cases and controls.

### THE EFFECT OF ALCOHOL ON DNA METHYLATION AT DIFFERENT LOCI (FAS CASES COMPARED TO UNAFFECTED CONTROLS)

Unadjusted and adjusted results are presented to assess potential differences in methylation percentages at different CpG sites and also across loci, between controls and FAS cases. The random variation between sites, individuals and replicates per individual was adjusted for in all analyses.

Table 6 gives a summary of the estimated differences in CpG methylation between FAS cases and controls (FAS-CON), per CpG site, unadjusted and adjusted for age and sex. Both models were adjusted for random variation between and within individuals.

**TABLE 6:** **Summary of estimated differential CpG methylation between FAS cases and controls (FAS-CON), per CpG site**.

		**Unadjusted**			**Adjusted for age and sex**		
**Locus**	**Site**	**FAS-CON**	**SE**	***P*-value**	**FAS-CON**	**SE**	***P*-value**
*H19 ICR*	CpG1	1.80	0.5	<0.001	0.23	0.79	0.767
*H19 ICR*	CpG2	1.06	0.5	0.035	–0.49	0.79	0.537
*H19 ICR*	CpG3	1.17	0.5	0.019	–0.42	0.79	0.594
*H19 ICR*	CpG5	1.48	0.5	0.003	–0.05	0.79	0.950
*H19 ICR*	CpG6	1.35	0.5	0.007	–0.16	0.79	0.835
*IG-DMR.A*	CpG1	1.02	0.53	0.054	–0.50	0.81	0.540
*IG-DMR.A*	CpG2	1.07	0.53	0.043	–0.52	0.81	0.520
*IG-DMR.A*	CpG3	0.48	0.53	0.364	–1.01	0.81	0.216
*IG-DMR.A*	CpG4	0.19	0.53	0.724	–1.27	0.81	0.117
*IG-DMR.A*	CpG5	3.05	0.53	<0.001	1.50	0.81	0.065
*IG-DMR.B*	CpG6	1.34	0.53	0.012	<0.21	0.81	0.792
*IG-DMR.B*	CpG7	0.22	0.53	0.679	–1.31	0.81	0.106
*IG-DMR.B*	CpG8	0.68	0.53	0.202	–0.92	0.81	0.258
*IG-DMR.B*	CpG9	1.36	0.53	0.010	–0.22	0.81	0.782
*IG-DMR.B*	CpG10	0.24	0.53	0.646	–1.33	0.81	0.101
*KvDMR1*	CpG1	0.96	0.53	0.072	–0.53	0.81	0.512
*KvDMR1*	CpG2	0.28	0.53	0.596	–1.21	0.81	0.138
*KvDMR1*	CpG3	0.28	0.53	0.595	–1.20	0.81	0.141
*KvDMR1*	CpG4	–0.17	0.53	0.752	–1.67	0.81	0.040
*KvDMR1*	CpG5	–0.01	0.53	0.986	–1.43	0.81	0.079
*KvDMR1*	CpG6	–0.10	0.53	0.851	–1.55	0.81	0.057
*KvDMR1*	CpG7	–0.60	0.53	0.262	–2.12	0.81	0.009
*PEG3 DMR*	CpG1	–5.34	0.55	<0.001	–6.98	0.83	<0.001
*PEG3 DMR*	CpG2	–5.08	0.55	<0.001	–6.69	0.83	<0.001
*PEG3 DMR*	CpG3	–6.14	0.55	<0.001	–7.73	0.83	<0.001
*PEG3 DMR*	CpG4	–4.74	0.55	<0.001	–6.43	0.83	<0.001
*PEG3 DMR*	CpG5	–5.51	0.55	<0.001	–7.07	0.83	<0.001
*PEG3 DMR*	CpG6	–5.11	0.55	<0.001	–6.67	0.83	<0.001
*PEG3 DMR*	CpG7	–5.35	0.55	<0.001	–6.96	0.83	<0.001

The analysis was unadjusted and adjusted for age and sex. Both models were adjusted for random variation between and within individuals.

At *H19 ICR*, all six sites, and at *IG-DMR* sites 2, 5, 6, and 9, the case group had significantly higher methylation than the control group. However after adjusting for age and sex there was no longer a significant difference between controls and cases. The only significant effects detected at *KvDMR1*, were at sites 4 and 7, where methylation was significantly lower in FAS cases than controls, after adjustment for age and sex. At *PEG3 DMR*, across all CpG sites, estimated methylation was very highly significantly lower (all *p*-values < 0.001) in FAS than in controls, with and without adjustment for age and sex.

The estimated methylation percentage difference between controls and cases across each locus is summarized in Table 7 and the observed percentage methylation is shown in Figure 2. At the *H19 ICR* locus, cases showed a highly significant increased average methylation compared to the controls, but this was no longer significant after adjusting for age and sex. At *KvDMR1* locus showed a significant lower average methylation after age and sex were adjusted. In the unadjusted analysis, the average methylation was significantly higher (1.15 and 0.75% respectively) in cases than controls, however after adjusting for age and sex the direction of the effect had changed but the reduced methylation was only significant at region B. The *PEG3 DMR* also showed a highly significant difference between cases and controls and the unadjusted (*p* < 0.001) and adjusted (*p* < 0.001) effect sizes were similar (5.47% lower in cases before adjustment and 7.09% lower in cases after adjustment).

**TABLE 7:** **Estimated differences in percentage methylation between cases and controls at each locus**.

	**Unadjusted**			**Adjusted for age and sex**		
**Locus**	**Effect**	**SE**	***P*-value**	**Effect**	**SE**	***P*-value**
*H19 ICR*	1.36	0.31	<0.001	–0.17	0.41	0.674
*IG-DMR1.A*	1.15	0.30	<0.001	–0.40	0.40	0.315
*IG-DMR1.B*	0.75	0.30	0.012	–0.84	0.40	0.035
*KvDMR1*	0.01	0.25	0.967	–1.49	0.37	<0.001
*PEG3 DMR*	–5.47	0.26	<0.001	–7.09	0.37	<0.001

SE, standard error. Significant: p < 0.05. Analyses were adjusted for variation between sites and variation between individuals, with and without adjustment for age and sex.

## DISCUSSION

Epigenetic modulation is increasingly studied as an important mechanism to explain fetal outcome based on environmental exposures during *in utero* development, with some effects lasting into adulthood. This includes maternal diet and exposure to teratogens, like alcohol, but may also include factors like stress. Since imprinted loci play an important role in fetal development, cellular differentiation and growth, we decided to investigate the levels of CpG methylation at four primary DMRs in children with FAS compared to methylation in unaffected controls. Our understanding of the relationship between DNA methylation with regard to sex, age and cell type remains incomplete, but in addition to inter-individual variation, it is clear that there are locus-specific effects. It is therefore expected that teratogens would also display locus-specific effects explaining their impact on fetal outcome. In addition, tissue-specific DNA methylation and tissue-specific epigenetic responses to prenatal alcohol exposure could potentially confound the interpretation of our study as we examined blood and buccal DNA from the participants, rather than neuronal tissue derived DNA.

### SEX AND AGE DEMONSTRATE LOCUS SPECIFIC METHYLATION EFFECTS ON SELECTED ICRs

The effect of sex on global DNA methylation and locus-specific methylation has been reported. Global DNA methylation has a tendency toward higher methylation levels in males (Fuke et al., 2004; Shimabukuro et al., 2007). Studies on the effect of sex on locus-specific methylation have shown both increases and decreases in DNA methylation (Sandovici et al., 2005; Sarter et al., 2005; Eckhardt et al., 2006; El-Maarri et al., 2007).

In this study, the effect of sex on methylation was shown to be significant at only one locus, *PEG3 DMR*. Interestingly the effects are modest, but opposite in FAS cases and controls, with the former showing increased methylation (1.11%) in males and the latter a decrease of 0.84% in males. It is not clear why the sex effect on methylation is different in the two groups, but it may be due to the fact that the data were not adjusted for age when the analysis was done because it was done as a baseline comparison to decide if sex needed to be adjusted for in the main analysis. *PEG3 DMR* average methylation was shown to decrease in controls for every 1 year increase in age, suggesting that there may be an age sex interaction at this locus. There was no effect of sex on average methylation at *H19 ICR*, *KvDMR1,* and *IG-DMR.*

Age is reported to cause a reduction in global DNA methylation and causes dramatic changes in the distribution of 5-methylcytosine across the genome (Liu et al., 2011). With respect to specific genes, methylation can either be increased or decreased depending on the gene investigated (as reviewed by Liu et al., 2003). Issa et al. (1996) reported that the *IGF2 P2-P4* promoter-associated CpG island is methylated on the silenced maternal allele in young individuals, however with age this methylation also appears on the paternal allele resulting in biallelic methylation (indicating an overall increase in methylation with age). The promoter regions of many genes tend to switch from an unmethylated to a methylated state resulting in gene silencing in an age dependent manner. This includes the promoters of several tumor and aging related genes (Wilson and Jones, 1983; Fuke et al., 2004; Liu et al., 2011). The mechanism contributing to the age dependent changes in global methylation includes a decrease in the expression of *DMNT1* (Lopatina et al., 2002; Liu et al., 2003). Longitudinal research on age effects that study the same individuals at several time points is rare (Florath et al., 2014; Flanagan et al., 2015). In two studies DNA methylation of participants was examined at two ages only, one where they were sampled 6 years apart and the other 8 years apart. It is therefore not yet clear whether age-related changes in methylation at CpG loci associated with age effects occur linearly with age.

We examined the effect of age on the different CpG sites and average methylation across each locus, separately in FAS cases and controls. In the control group, with the exception of *PEG3 DMR* CpG7, there was no CpG site specific age effect. In the FAS cases however, eight out of the 10 *IG-DMR* CpG sites, one *KvDMR1* site and one *PEG3 DMR* site showed a significant age effect. With a single exception, methylation in the FAS group decreased by a modest amount for every additional year of age. When examining the locus-averaged methylation and the effect of age, there was a small but significant effect for *KvDMR1*, but a larger effect in the FAS cases for *IG-DMR* (for both region A and B). This effect was not observed in controls. In contrast, the controls showed an age effect at the *PEG3 DMR*. The measure for an age effect is “difference in methylation per additional year of age”; however there was no overlap in absolute age between cases and controls. From our results, it would appear that age effects are more significant at younger ages (1–16 years) than in older age groups (17–26), in a locus-specific manner.

In this study age was shown to influence methylation at three of the four loci investigated. In alignment with our findings, a study on periconceptional famine exposure (Heijmans et al., 2008) found that within the age group of 43–70 years, the DNA methylation at the *IGF2 DMR* of a 10 year older group was associated with a 3.6% lower methylation (*p* = 0.015) in controls. The magnitude (0.36% per annum) of the effect in their study was greater than that observed in our study.

Since both sex and age showed some effect on DNA methylation at one or more of the imprinted loci in this study, we present sex and age adjusted analyses when comparing DNA CpG methylation between FAS cases and unaffected controls.

### THE EFFECT OF *IN UTERO* ALCOHOL EXPOSURE ON DNA METHYLATION AT FOUR IMPRINTED LOCI

We assessed the possible effect of maternal alcohol consumption on DNA methylation at *H19 ICR*, *KvDMR1*, *IG-DMR*, and *PEG3 DMR*, by comparing methylation levels between FAS cases and unaffected controls. After adjustment for sex and age there was no observed correlation with *in utero* alcohol exposure at the CpG site level at two of the imprinted loci, *H19 ICR* and *IG-DMR*. Interestingly, a modest effect (*p* = 0.035) of decreased methylation (0.84%) for *IG-DMR* Region B was observed in FAS cases. The *IG-DMR* Region B shows roughly 50% methylation, in line with a parent of origin allelic effect whereas Region A had an overall higher methylation percentage.

The *IG-DMR* is a good candidate in terms of its potential biological impact, in line with the features of FAS. The paternally methylated *IG-DMR* is the primary ICR at the *DLK1/GTL2* (*MEG3*) imprinting domain on human chromosome 14q32, where it plays an essential role in regulating the monoallelic expression of several imprinted genes including the paternally expressed *DLK1* and maternally expressed *GTL2* genes (Lin et al., 2003). The methylation on the paternal allele is essential in maintaining the expression of imprinted genes, because failure to maintain the paternal methylation has been shown to result in considerable *Dlk* repression while *Gtl2* expression is increased (Schmidt et al., 2000).

The *DLK1/GTL2* (*MEG3*) imprinting cluster is a critical region for the phenotypes associated with both maternal and paternal uniparental disomy (UPD) of chromosome 14 (Coveler et al., 2002; Kagami et al., 2005; Temple et al., 2007; Buiting et al., 2008). Maternal uniparental disomy 14 [Upd(14)mat] and hypomethylation at the paternally imprinted *IG-DMR* (Ogata et al., 2008) are characterized by pre- and postnatal growth retardation, developmental delays, mild to moderate mental retardation, muscular hypotonia, small hands and feet, premature puberty and truncal obesity. The locus-averaged methylation of the *IG-DMR* was modestly reduced in FAS cases, tending toward hypomethylation and which may potentially contribute to the growth and neuronal deficits in affected individuals. The magnitude of alcohol effects may be tissue specific and may play a more important role in neurogenesis. These findings merit further study and validation.

After adjustment of sex and age, two *KvDMR1* CpG sites (4 and 7) showed significantly decreased DNA methylation in FAS cases which contributed to a locus-averaged decrease of 1.49% methylation in the *KvDMR1*. The functional impact of this difference is not clear. The biggest effect (a decrease of 7.09% methylation in FAS cases) was observed at the *PEG3 DMR*. Interestingly, it is the two maternally imprinted loci, *KvDMR1* and *PEG3 DMR*, which are significantly affected by *in utero* alcohol exposure and both show a decrease in methylation following alcohol exposure.

One of the key features of FAS is pre- and post-natal growth retardation and dysregulation of imprinting at *H19 ICR* has been associated with growth disorders (Reik et al., 1995; Gicquel et al., 2005; Ideraabdullah et al., 2008). The findings of our study are, however, in agreement with a study done in a mouse model by Haycock and Ramsay (2009) where they reported no difference in methylation at the *H19 ICR* of mouse embryos exposed to alcohol during the preimplantation period, when compared to unexposed control embryos. Interestingly *H19 ICR* hypomethylation was observed in the mouse placentas suggesting a localized effect on the extra-embryonic tissue, which could explain the effect on fetal growth. In two other related studies subtle differential DNA methylation was observed. Knezovich and Ramsay (2012) reported a significant decrease in methylation at the *H19 ICR* in mouse offspring following preconception paternal alcohol exposure and Downing et al. (2011) reported a subtle decrease in methylation at the mouse *Igf2 DMR1* locus in embryos following *in utero* alcohol exposure.

The hypomethylation at *KvDMR1* and *PEG3 DMR* is aligned to our original hypothesis suggesting that alcohol reduces DNA methylation through the one carbon metabolism pathway and its effect on reducing folate levels. In the next sections the potential implications of hypomethylation at these loci are explored.

### THE FUNCTIONAL IMPACT OF REDUCED *KvDMR1* METHYLATION IN FAS CASES IS UNCLEAR

*KvDMR1* CpG site-specific and average locus-wide hypomethylation in response to *in utero* alcohol exposure would suggest a loss of methylation on the maternally methylated ICR which regulates the monoallelic expression of several imprinted genes located in the *CDKN1C*/*KCNQ1OT1* imprinting domain cluster. This imprinting domain harbors the paternally expressed non-coding antisense transcript to *KCNQ1* called *KCNQ1OT1*, and other maternally expressed protein coding genes including *KCNQ1* and *CDKNIC1*. Loss of imprinting, or hypomethylation, at the *KvDMR1* has been widely implicated in the Beckwith–Wiedemann syndrome (BWS; Gaston et al., 2001; Diaz-Meyer et al., 2003; Azzi et al., 2009), a congenital disorder characterized by pre- and postnatal overgrowth, organomegaly, and a high risk of childhood tumors (Weksberg et al., 2010). Paradoxically, the FAS cases showed significant hypomethylation at CpG sites 4 and 7 (1.67 and 2.1%, respectively), yet FAS affected individuals are growth restricted. It is unclear whether hypomethylation of only two of the seven CpG sites in this ICR will affect the levels of expression of the imprinted genes in the cluster and what the functional effect may be.

To gain further insight into gene regulation at this locus will require both gene expression and DNA methylation studies to more fully understand the impact of altered methylation at the *KvDMR1*. This is the first study to show the effect of alcohol on methylation status at *KvDMR1* and the findings are counter intuitive given that hypomethylation is associated with an overgrowth phenotype (BWS).

### UNDERSTANDING THE ROLE OF ALCOHOL INDUCED HYPOMETHYLATION AT THE *PEG3* IMPRINTED GENE CLUSTER IN THE PATHOGENESIS OF FAS REQUIRES FURTHER KNOWLEDGE OF THE ICR CONTROLLED GENE EXPRESSION IN THIS REGION

The *PEG3* imprinting cluster is located on human chromosome 19q13.4 and is regulated by a maternally methylated ICR, the *PEG3 DMR*. The cluster includes several imprinted genes including the paternally expressed 3 gene (*PEG3*), the imprinted zinc-finger gene 2 (*ZIM2*) gene and the *USP29* gene, all of which are paternally expressed. Although these loci are syntenic in mouse and human, there are some interesting differences regarding their regulation, their tissue specific expression, and their exon structure and genomic arrangement. The *PEG3* gene is expressed in embryonic tissues, including the hypothalamus and brain, and in adult mouse and human brain, but most highly in human ovary, but not mouse ovary. *PEG3* encodes a DNA binding protein based on its multiple zing finger motifs (Relaix et al., 1996; Iuchi, 2001) and is an imprinted transcription factor that has multiple target genes (Thiaville et al., 2013). It has a proposed tumor suppressive function (Nye et al., 2013) and has been shown to induce *p53*-mediated apoptosis in multiple cell types (Yamaguchi et al., 2002). A mouse knockout model targeting the *Peg3* gene has shown that it is responsible for a variety of phenotypic outcomes including altered maternal offspring rearing behavior, low birth weight, alteration in fat tissue storage and synthesis, and lower metabolic activity (Li et al., 1999; Curley et al., 2004).

We observed that maternal alcohol consumption is correlated with a significant reduction of ∼7% methylation at the *PEG3 DMR* in FAS cases. The highly significant decrease in methylation was observed for all the CpG sites analyzed for this locus and also for the average methylation across this locus. It is possible that this change in the *PEG3 ICR* may affect multiple imprinted genes in the region. *PEG3* is expressed from the paternal allele and is reciprocally repressed on the maternal allele, suggesting that alcohol induced demethylation likely affects the maternal allele thus leading to derepression of the *PEG3* gene on the maternal allele, and therefore biallelic expression of *PEG3*. This would lead to an overall increase in *PEG3* expression. Several studies have focussed on the effects of reduced *Peg3*, but none has explored the phenotypic outcome of over expression of *PEG3*.

Gene expression studies, without correlation to their imprinting status, have demonstrated upregulation of *PEG3* (as well as several other genes) in intrauterine growth restriction (IUGR) placentas (reviewed in Ishida and Moore, 2013). Since IUGR is a cause of reduced fetal growth, this study supports our finding that the proposed increase in *PEG3* expression could be associated with a growth restriction phenotype. The role of the *PEG3 DMR* in regulating the imprinted gene cluster in humans requires further investigation.

## CONCLUSION

Despite limitations in the study design, including the lack of age matching between cases and controls, the relatively small sample size, and the inaccessibility of neuronal tissue, significant differences in DNA methylation were observed at two primary DMRs when comparing FAS cases with unaffected controls. The observed hypomethylation at the *KvDMR1* has uncertain functional impact on gene expression and the FAS phenotype. The largest epigenetic effect among the loci investigated, was a locus-averaged 7% reduction in DNA methylation at the *PEG3 DMR* which was observed across all seven CpG sites. This ICR orchestrates a complex pattern of gene expression across the region with reported differences in mouse models compared to humans. It is proposed that hypomethylation of the *PEG3 DMR* would result in an increase in the paternally expressed *PEG3* gene. *PEG3* has a DNA binding motif and is considered an imprinted transcription factor, and therefore its function is most likely mediated by altered expression of its targets. Although there is some spatiotemporal congruence of gene expression in line with the developmental origins of the FAS related phenotype, the effect and mechanism of altered expression of *PEG3* and the other imprinted genes controlled by the *PEG3 DMR* remains unclear. Despite the uncertainty of the functional biological mechanism of the locus-specific hypomethylation of important ICRs in the blood of FAS cases, these findings support the role of an epigenetic mechanism in the development of FAS.

### Conflict of Interest Statement

The authors declare that the research was conducted in the absence of any commercial or financial relationships that could be construed as a potential conflict of interest.
